# Chemical Probes that Competitively and Selectively Inhibit Stat3 Activation

**DOI:** 10.1371/journal.pone.0004783

**Published:** 2009-03-10

**Authors:** Xuejun Xu, Moses M. Kasembeli, Xueqing Jiang, Benjamin J. Tweardy, David J. Tweardy

**Affiliations:** 1 Section of Infectious Diseases, Department of Medicine, Baylor College of Medicine, Houston, Texas, United States of America; 2 Department of Molecular and Cellular Biology, Baylor College of Medicine, Houston, Texas, United States of America; Monash University, Australia

## Abstract

Signal transducer and activator of transcription (Stat) 3 is an oncogene constitutively activated in many cancer systems where it contributes to carcinogenesis. To develop chemical probes that selectively target Stat3, we virtually screened 920,000 small drug-like compounds by docking each into the peptide-binding pocket of the Stat3 SH2 domain, which consists of three sites—the pY-residue binding site, the +3 residue-binding site and a hydrophobic binding site, which served as a selectivity filter. Three compounds satisfied criteria of interaction analysis, competitively inhibited recombinant Stat3 binding to its immobilized pY-peptide ligand and inhibited IL-6-mediated tyrosine phosphorylation of Stat3. These compounds were used in a similarity screen of 2.47 million compounds, which identified 3 more compounds with similar activities. Examination of the 6 active compounds for the ability to inhibit IFN-γ-mediated Stat1 phosphorylation revealed that 5 of 6 were selective for Stat3. Molecular modeling of the SH2 domains of Stat3 and Stat1 bound to compound revealed that compound interaction with the hydrophobic binding site was the basis for selectivity. All 5 selective compounds inhibited nuclear-to-cytoplasmic translocation of Stat3, while 3 of 5 compounds induced apoptosis preferentially of breast cancer cell lines with constitutive Stat3 activation. Thus, virtual ligand screening of compound libraries that targeted the Stat3 pY-peptide binding pocket identified for the first time 3 lead compounds that competitively inhibited Stat3 binding to its pY-peptide ligand; these compounds were selective for Stat3 vs. Stat1 and induced apoptosis preferentially of breast cancer cells lines with constitutively activated Stat3.

## Introduction

Signal transducer and activator of transcription 3 (Stat3) is an oncogene [1] and one of seven members of the Stat protein family, which are signaling intermediates that mediate the actions of many cytokines and growth factors. Stat3 is constitutively active in many different cancers including prostate, breast, lung, head and neck, colon, liver, and pancreas as well as in multiple myeloma and large granular lymphocytic leukemia [2]–[8]. Furthermore, human tumor xenograft studies in mice have repeatedly demonstrated that inhibiting Stat3 results in decreased tumor growth and improved animal survival [4] by inducing apoptosis in tumor cells, inhibiting angiogenesis [9] and enhancing anti-tumor immune-mediated cytotoxicity [2], [10]. Thus, Stat3 has been identified as a potentially high-yield target for drug development to treat many cancers [11]–[13].

In contrast to Stat3, Stat1 is anti-oncogenic; it is a potent inhibitor of tumor growth and promoter of apoptosis [1]. Also, because tumors from carcinogen-treated wild-type animals grow more rapidly when transplanted into the Stat1-deficient animals than they do in a wild-type host, Stat1 contributes to tumor immunity [14]. Consequently, a highly desirable goal in the development of drugs that target Stat3 is selectivity for Stat3 vs. Stat1.

We and others have developed drugs that selectively target Stat3 vs. Stat1[15]–[20]. However, determination of their selectivity was established empirically after their identification as Stat3 inhibitors and was not built into the screening process. In this paper, we describe a small-molecule, virtual ligand screening approach that targets the pY-peptide binding pocket of the Stat3 SH2 domain at three sites including a hydrophobic pocket, which served as a selectivity filter. This approach identified for the first time 3 novel lead compounds that competitively inhibit Stat3 binding to its pY-peptide ligand, that are selective for Stat3 vs. Stat1 and that also induce apoptosis preferentially of breast cancer cells lines with constitutively activated Stat3. In addition to yielding compounds that selectively target Stat3 by design, the approach described has potential for identifying selective, chemical probes of other members of the Stat protein family.

## Methods

### Virtual ligand screening

We isolated the three-dimensional structure of the Stat3 SH2 domain from the core fragment structure of phosphorylated Stat3 homodimers bound to DNA [21] deposited in the RCSB Protein Data Bank (PDB) databank (PDB code 1BG1) and converted it to be an Internal Coordinate Mechanics (ICM)-compatible system by adding hydrogen atoms, modifying unusual amino acids, making charge adjustments and performing additional cleanup steps. In addition, we retrieved the coordinates of the Stat1 SH2 domain from the PDB databank (PDB code 1BF5) for use in computational selectivity analysis [22]. Commercial chemical databases (ChemBridge, Asinex, ChemDiv, Enamine, KeyOrganics and LifeChemicals) were chosen as sources of compounds for screening *in silico*. We selected the amide hydrogen of E638 within the site that binds the +3 residue (Q, C or T) within the pY-peptide ligand [23] as the central point of the binding pocket, which consisted of a cube with dimensions 16.0×16.9×13.7 angstrom. In addition to the +3 binding site, this cube contained the pY residue binding site consisting mainly of R609 and K591 [23] and a hydrophobic binding site consisting of 5 residues—W623, Q635, V637, Y640 and Y657. Alignment of the residues of Stat3 from W623 to Y657 that contain the hydrophobic binding site and the corresponding residues of Stat1 revealed a difference in 1 of these 5 residues (Q635 in Stat3 vs. H629 in Stat1). In addition, there was only 40% homology in the remaining residues within this region. Also, overlay of the Stat3 and Stat1 SH2 domain peptide backbone structures did not reveal superimposition throughout this region, particularly within Loop_βC–βD_ (K_626_DISGSTQIQS_636_). Finally, comparison of the orientation of the hydrophobic binding site residues revealed that the side chain of V637 in Stat3 is pointed into the hydrophobic binding pocket while the corresponding residue V631 in Stat1 is pointed away from the pocket. These considerations raised the possibility that the hydrophobic binding site might serve as a selectivity filter [24]. A flexible docking calculation [25] was performed in order to determine the global minimum energy score and thereby predict the optimum conformation of the compound within the pocket. A compound was selected for purchase and biochemical testing based on fulfilling the criteria of interaction analysis (CIA): 1) global minimum energy score ≤−30; 2) formation of a salt-bridge and/or H-bond network within the pY-residue binding site; and 3) formation of an H-bond with or blocking access to the amide hydrogen of E638. Most, but not all, compounds also interacted with the hydrophobic binding site.

### Stat3/pY-peptide binding assay

Stat3 binding assays were performed at 25°C with a BIAcore 3000 biosensor using 20 mM Tris buffer pH 8 containing 2 mM mercaptoethanol and 5% DMSO as the running buffer [26]. Phosphorylated and control non-phosphorylated biotinylated EGFR derived dodecapeptides based on the sequence surrounding Y1068 [27] were immobilized on a streptavidin coated sensor chip (BIAcore inc., Piscataway NJ). The binding of Stat3 was conducted in 20 mM Tris buffer pH 8 containing 2 mM ß-mercaptoethanol at a flow rate of 10 µL/min for 1–2 minute. Aliquots of Stat3 at 500 nM were premixed with compound to achieve a final concentration of 1–1,000 µM and incubated at 4°C prior to being injected onto the sensor chip. The chip was regenerated by injecting 10 µL of 100 mM glycine at pH 1.5 after each sample injection. A control (Stat3 with DMSO but without compound) was run at the beginning and the end of each cycle (40 sample injections) to ensure that the integrity of the sensor chip was maintained throughout the cycle run. The average of the two controls was normalized to 100% and used to evaluate the effect of each compound on Stat3 binding. Responses were normalized by dividing the value at 2 min by the response obtained in the absence of compounds at 2 min and multiplying by 100. IC_50_ values were determined by plotting % maximum response as a function of log concentration of compound and fitting the experimental points to a competitive binding model using a four parameter logistic equation: R = R_high_−(R_high_−R _low_)/ (1+conc/A1)^A2^, where R = percent response at inhibitor concentration, R_high_ = percent response with no compound, R_low_ = percent response at highest compound concentration, A2 = fitting parameter (slope) and A1 = IC_50_ (BIAevaluation Software version 4.1).

### Immunoblot assay

The human hepatocellular carcinoma cell line (HepG2) was grown in 6-well plates under standard conditions. Cells were pretreated with compounds (0, 1, 3, 10, 30, 100 and 300 µM) for 1 hour then stimulated under optimal conditions with either interleukin-6 (IL-6; 30 ng/ml for 30 min) to activate Stat3 or interferon gamma (IFN-γ; 30 ng/ml for 30 min) to activate Stat1 [28]. Cultures were then harvested and proteins extracted using high-salt buffer, as described [23]. Briefly, extracts were mixed with 2× sodium dodecyl sulfate (SDS) sample buffer (125 mM Tris-HCL pH 6.8; 4% SDS; 20% glycerol; 10% 2-mercaptoethanol) at a 1∶1 ratio and heated for 5 minutes at 100°C. Proteins (20 µg) were separated by 7.5% SDS-PAGE and transferred to polyvinylidene fluoride (PVDF) membrane (Millipore, Waltham, MA) and immunoblotted. Prestained molecular weight markers (BioRad; Hercules, CA) were included in each gel. Membranes were probed serially with antibody against Stat1 pY701 or Stat3 pY705 followed by antibody against Stat1 or Stat3 (Transduction labs, Lexington, KY) then antibody against β–actin (Abcam, Cambridge, MA). Membranes were stripped between antibody probing using Restore™ Western Blot Stripping Buffer (Thermo Fisher Scientific Inc., Waltham, MA) per the manufacturer's instructions. Horseradish peroxidase-conjugated goat-anti-mouse IgG was used as the secondary antibody (Invitrogen Carlsbad, CA) and the membranes were developed with enhanced chemiluminescence (ECL) detection system (Amersham Life Sciences Inc.; Arlington Heights, IL.).

### Similarity screen

Three compounds identified in the initial VLS—Cpd3, Cpd30 and Cpd188—inhibited Stat3 SH2/pY-peptide binding and IL-6-mediated Stat3 phosphorylation and were chosen as reference molecules for similarity screening. A fingerprint similarity query for each reference compound was submitted to Molcart/ICM (Max Distance, 0.4). Similarity between each reference molecule and each database molecule was computed and the similarity results were ranked in decreasing order of ICM similarity score [29]. The databases searched included ChemBridge, LifeChemicals, Enamine, ChemDiv, Asinex, AcbBlocks, KeyOrganics and PubChem for a total of 2.47 million compounds. All compounds identified were docked into the binding pocket of Stat3 SH2 domain *in silico*. Compounds that fulfilled CIA criteria were purchased and tested as described for compounds identified in the primary screen.

### Molecular modeling

All 3-D configurations of the Stat3 SH2 domain complexed with compounds were determined by global energy optimization that involves multiple steps: 1) location of organic molecules were adjusted as a whole in 2 Å amplitude by pseudo-Brownian random translations and rotations around the molecular center of gravity, 2) the internal variables of organic molecules were randomly changed, 3) coupled groups within the Stat3 SH2 domain side-chain torsion angles were sampled with biased probability shaking while the remaining variables of the protein were fixed, 4) local energy minimizations were performed using the Empirical Conformation Energy Program for Peptides type-3 (ECEPP3) in a vacuum [30] with distance-dependent dielectric constant є = 4r, surface-based solvent energy and entropic contributions from the protein side chains evaluated added and 5) conformations of the complex, which were determined by Metropolis criteria, were selected for the next conformation-scanning circle.

The initial 3-dimensional configuration of the Stat1 SH2 domain in a complex with each compound was predicted and generated by superimposing, within the computational model, the 3-dimensional features of the Stat1 SH2 domain onto the 3-dimensional configuration of the Stat3 SH2 domain in a complex with each compound. The peptide backbone atoms of residues K584, R602 and E632 in Stat1 and K591, R609 and E638 in Stat3 were used in this superimposition. The final computational model of Stat1 SH2 in a complex with each compound was determined by local minimization using Internal Coordinate Force Field (ICFF)-based molecular mechanics [25]. We computed the van der Waals energy of each complex consisting of compound bound to the SH2 domain of Stat1 or Stat3 using Lennard-Jones potential with ECEPP/3 force field [30].

### Confocal and high-throughput fluorescence microscopy (HTFM)

Confocal and high-throughput fluorescence microscopy (HTFM) of MEF/GFP-Stat3α cells were performed as described [31]. Briefly, for confocal fluorescence microscopy, cells were grown in 6-well plates containing a cover slip. For HTFM, cells were seeded into 96-well CC3 plates at a density of 5,000 cells/well using an automated plating system. Cells were cultured under standard conditions until 85–90% confluent. Cells were pre-treated with compound for 1 hour at 37°C then stimulated with IL-6 (200 ng/ml) and IL-6sR (250 ng/ml) for 30 minutes to provide optimal Stat3 activation and nuclear translocation in these cells, as described [31]. Cells were fixed with 4% formaldehyde in PEM Buffer (80 mM Potassium PIPES, pH 6.8, 5 mM EGTA pH 7.0, 2 mM MgCl2) for 30 minutes at 4°C, quenched in 1 mg/ml of NaBH4 (Sigma) in PEM buffer and counterstained for 1 min in 4,6-diamidino-2-phenylindole (DAPI; Sigma; 1 mg/ml) in PEM buffer. Cover slips were examined by confocal fluorescent microscopy. Plates were analyzed by automated HTFM using the Cell Lab IC Image Cytometer (IC100) platform and Cytoshop Version 2.1 analysis software (Beckman Coulter). Nuclear translocation is quantified by using the fraction localized in the nucleus (FLIN) measurement [32]. FLIN values were normalized by subtracting the FLIN for unstimulated cells then dividing this difference by the maximum difference (delta, Δ) in FLIN (FLIN in cells stimulated with IL-6/sIL-6R in the absence of compound minus FLIN of unstimulated cells). This ratio was multiplied by 100 to obtain the percentage of maximum difference in FLIN and was plotted, where indicated, as a function of the log compound concentration. The best-fitting curve and IC_50_ value were determined using 4-Parameter Logistic Model/Dose Response/XLfit 4.2, IDBS software.

### Breast cancer cell line apoptosis assay

Human breast carcinoma cell lines MDA-MB-468, MDA-MB-231, MBA-MD-435 and MCF7 were kindly provided by Dr. Powel H. Brown (Breast Cancer Center, Baylor College of Medicine). Breast cancer cell line, MDA-MB-453 was kindly provided by Dr. Shou Jiang (Breast Cancer Center, Baylor College of Medicine). All cell lines were grown in DMEM medium supplemented with 10% fetal bovine serum (FBS), 25,000 units penicillin G, 25,000 µg streptomycin, and 131.4 mg L-Glutamine and cultured in the incubator under the condition of 95% air, 5% CO2 at 37°C [33]. Cells were seeded at 2,500 cells/cm^2^ into 12-well plates. At 80% confluency, cells were washed with PBS and supplemented with fresh medium containing compound or the topoisomerase I-inhibitor, camptothecin, at 0, 0.1, 0.3, 1, 3, 10, 30, 100, 300 µM. At 24 hours, treatment was terminated by removing the medium from each well. Cells were lysed with cell lysis buffer (600 µl for 30 minutes at 25°C). Cell lysate (200 µl) was centrifuged at 200 g for 10 minutes and 20 µl of each supernatant was assayed for nucleosomes using the Cell Death Detection ELISA (Roche Applied Science) as described by the manufacturer. The percent maximum nucleosome level was calculated by dividing the nucleosome level by the maximum nucleosome level achieved in the assay and multiplying by 100. This value was plotted as a function of the log compound concentration and the best-fitting curve and EC_50_ value determined using 4-Parameter Logistic Model/Dose Response/XLfit 4.2, IDBS software.

## Results

### Identification by virtual ligand screening (VLS) of compounds that blocked Stat3 binding to its phosphopeptide ligand and inhibited IL-6-mediated phosphorylation of Stat3

**Figure 1:**
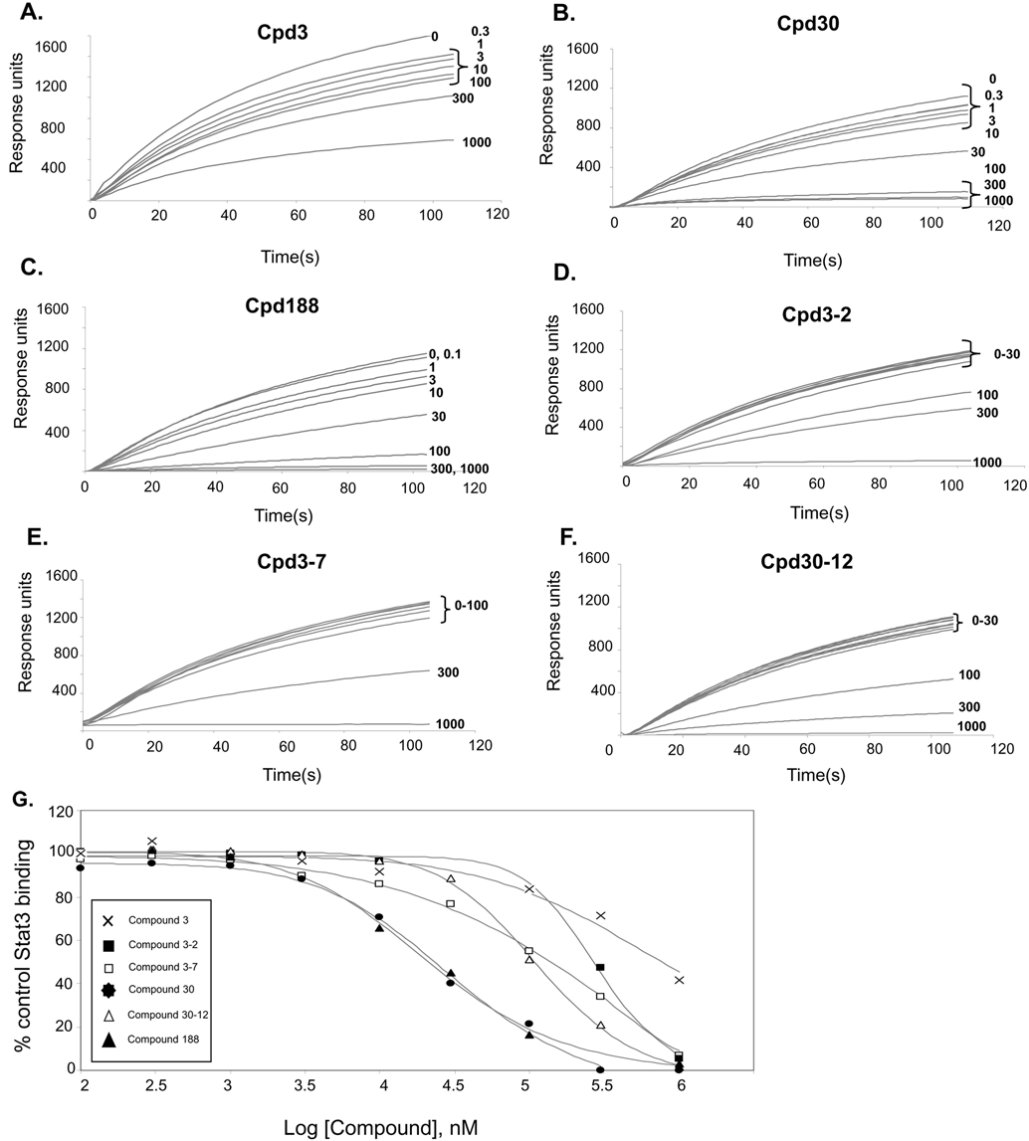
Inhibition of Stat3 binding to immobilized phosphopeptide ligand by compounds. Binding of recombinant Stat3 (500 nM) to a BiaCore sensor chip coated with a phosphododecapeptide based on the amino acid sequence surrounding Y1068 within the EGFR was measured in real time by SPR in the absence (0 µM) or presence of increasing concentrations (0.1 to 1,000 µM) of Cpd3 (panel A), Cpd30 (panel B), Cpd188 (panel C), Cpd3-2 (panel D), Cpd3-7 (panel E) and Cpd30-12 (panel F). Data shown are response units as a function of time in seconds and are representative of 2 or more experiments. The equilibrium binding levels obtained in the absence or presence of compound were normalized (response obtained in the presence of compound ÷ the response obtained in the absence of compound ×100) and plotted against the log concentration (nM) of the compound (panel G). The experimental points for each compound fit to a competitive binding curve that uses a four-parameter logistic equation (see Methods for details). These curves were used to calculate the IC_50_ value for each compound (Table 2).

**Figure 2:**
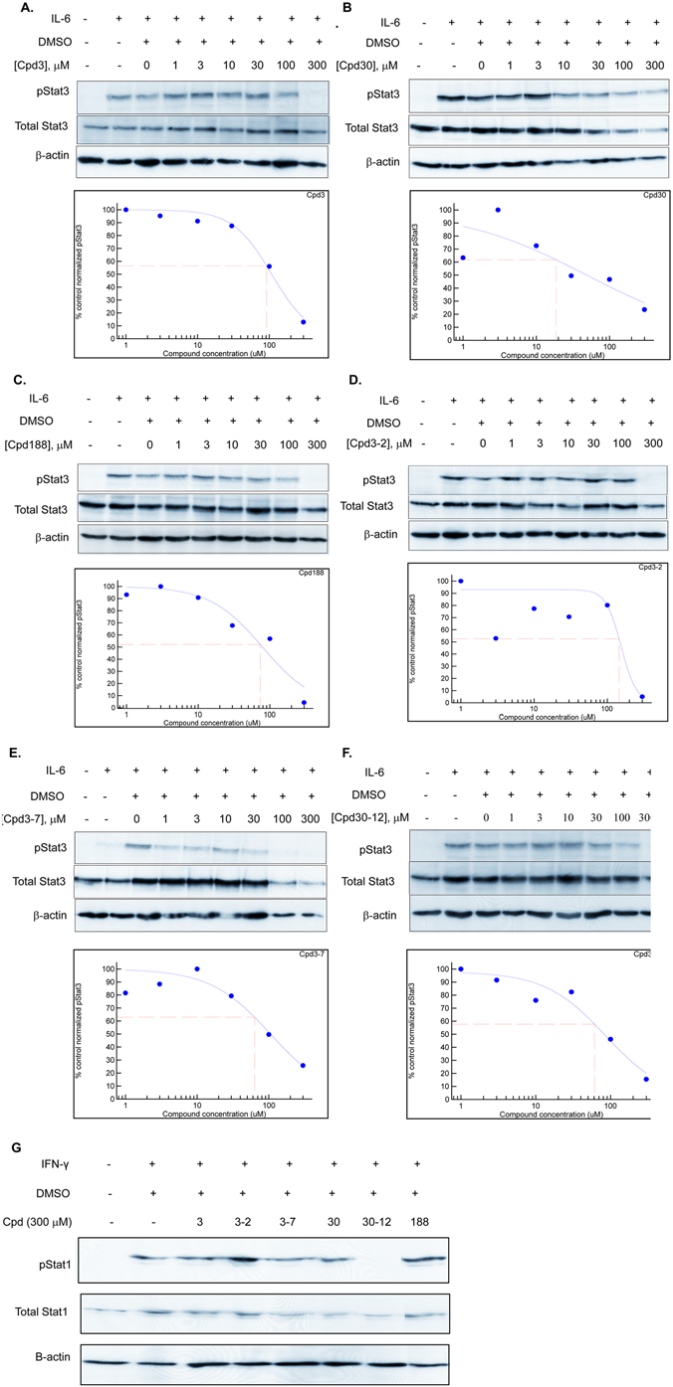
Effect of compounds on ligand-mediated Stat3 and Stat1 phosphorylation. HepG2 cells were pretreated with DMSO alone or DMSO containing Cpd3 (panel A), Cpd188 (panel B), Cpd30 (panel C), Cpd3-2 (panel D), Cpd3-7 (panel E) or Cpd30-12 (panel F) at the indicated concentration for 60 min. Cells were then stimulated with IL-6 (30 ng/ml) for 30 min. Protein extracts of cells were separated by SDS-PAGE, blotted and developed serially with antibodies to pStat3, total Stat3 and β-actin. Blots were stripped between each antibody probing. Band intensities were quantified by densitometry. The value of each pStat3 band was divided by its corresponding total Stat3 band intensity; the results were normalized to the DMSO-treated control value. This value was plotted as a function of the log compound concentration. The best-fitting curve was determined using 4-Parameter Logistic Model/Dose Response/XLfit 4.2, IDBS software and was used to calculate the IC_50_ value (Table 1). Each panel is representative of 3 or more experiments. In panel G, HepG2 cells were pretreated with DMSO alone or DMSO containing each of the compounds at a concentration of 300 µM for 60 min. Cells were then stimulated with IFN-γ (30 ng/ml) for 30 min. Protein extracts of cells were separated by SDS-PAGE and immunoblotted serially with antibodies to pStat1, total Stat1 and β-actin. Blots were stripped between each immunoblotting. The results shown are representative of 2 or more experiments.

Our VLS protocol was used to evaluate a total of 920,000 drug-like compounds. Of these, 142 compounds fulfilled CIA criteria. These compounds were purchased and tested for their ability to block Stat3 binding to its phosphopeptide ligand in a surface plasmon resonance (SPR)-based binding assay and to inhibit IL-6-mediated phosphorylation of Stat3. SPR competition experiments showed that of the 142 compounds tested, 3 compounds—Cpd3, Cpd30 and Cpd188—were able to directly compete with pY-peptide for binding to Stat3 with IC_50_ values of 447, 30, and 20 µM, respectively (Figure 1; Tables 1 and 2). In addition, each compound inhibited IL-6-mediated phosphorylation of Stat3 with IC_50_ values of 91, 18 and 73 µM respectively (Figure 2; Table 2).

**Table 1:** Summary of compound chemical names and formulas.

Compound^1^	Chemical Name	Formula
**Cpd3**	4-[3-(2,3-dihydro-1,4-benzodioxin-6-yl)-3-oxo-1-propen-1-yl]benzoic acid	C_18_H_14_O_5_
**Cpd30**	4-{5-[(3-ethyl-4-oxo-2-thioxo-1,3-thiazolidin-5-ylidene)methyl]-2-furyl}benzoic acid	C_17_H_13_NO_4_S_2_
**Cpd188**	4-[({3-[(carboxymethyl)thio]-4-hydroxy-1-naphthyl}amino)sulfonyl]benzoic acid	C_19_ H_15_ N O_7_ S_2_
**Cpd3-2**	3-({2-chloro-4-[(1,3-dioxo-1,3-dihydro-2H-inden-2-ylidene)methyl]-6-ethoxyphenoxy}methyl)benzoic acid	C_26_ H_19_ Cl O_6_
**Cpd3-7**	methyl 4-({[3-(2-methoxy-2-oxoethyl)-4,8-dimethyl-2-oxo-2H-chromen-7-yl]oxy}methyl)benzoate	C_23_ H_22_ O_7_
**Cpd30-12**	4-chloro-3-{5-[(1,3-diethyl-4,6-dioxo-2-thioxotetrahydro-5(2H)-pyrimidinylidene)methyl]-2-furyl}benzoic acid	C_20_ H_17_ Cl N_2_ O_5_ S

1 Compound name given by our lab.

**Table 2:** Summary of activity of compounds in inhibiting Stat3 binding to pY peptide ligand in a surface plasmon resonance binding (SPR) assay, in inhibiting IL-6-mediated Stat3 phosphorylation (pStat3) and in inhibiting IL-6-mediated Stat3 nuclear translocation in a high-throughput fluorescence microscopy (HTFM) assay.

Assay	Cpd3	Cpd30	Cpd188	Cpd3-2	Cpd3-7	Cpd30-12
**SPR**	447^1^	30	20	256	137	114
**pStat3**	91	18	73	144	63	60
**HTFM**	131	77	39	150	20	>300

1 Data presented are IC_50_ values (µM) obtained using results summarized in Figures 1 (SPR), Figure 2 (pStat3) and Figure 4 (HTFM).

Similarity screening with Cpd3, Cpd30 and Cpd188 identified 4,302 additional compounds. VLS screening was performed with each of these compounds, which identified 41 compounds that fulfilled CIA criteria; these were purchased and tested. SPR competition experiments showed that of these 41 compounds, 3 compounds—Cpd3-2, Cpd3-7 and Cpd30-12—were able to directly compete with pY-peptide for binding to Stat3 with IC_50_ values of 256, 137 and 114 µM, respectively (Figure 1; Tables 1 and 2). In addition, each compound inhibited IL-6-mediated phosphorylation of Stat3 with IC_50_ values of 144, 63 and 60 µM, respectively (Figure 2; Table 2).

### Compound-mediated inhibition of ligand-stimulated phosphorylation of Stat3 is specific for Stat3 vs. Stat1

While Stat3 contributes to oncogenesis, in part, through inhibition of apoptosis, Stat1 is anti-oncogenic; it mediates the apoptotic effects of interferons and contributes to tumor immunity [14], [34]. Consequently, compounds that target Stat3 while sparing Stat1, leaving its anti-oncogenic functions unopposed, may result in a synergistic anti-tumor effect. To assess the selectivity of our compounds for Stat3 vs. Stat1, HepG2 cells were incubated with Cpd3, Cpd30, Cpd188, Cpd3-2, Cpd3-7, and Cpd30-12 (300 µM) for 1 hour at 37°C before IFN-γ stimulation (Figure 2G). Only treatment with Cpd30-12 blocked Stat1 phosphorylation while each of the other five compounds—Cpd3, Cpd30, Cpd188, Cpd3-2 and Cpd3-7—did not. Thus, five of the six compounds identified were selective and inhibited ligand-stimulated phosphorylation of Stat3 but not Stat1.

### Sequence analysis and molecular modeling of the interaction of each compound with the Stat3 vs. Stat1 SH2 domain

**Figure 3:**
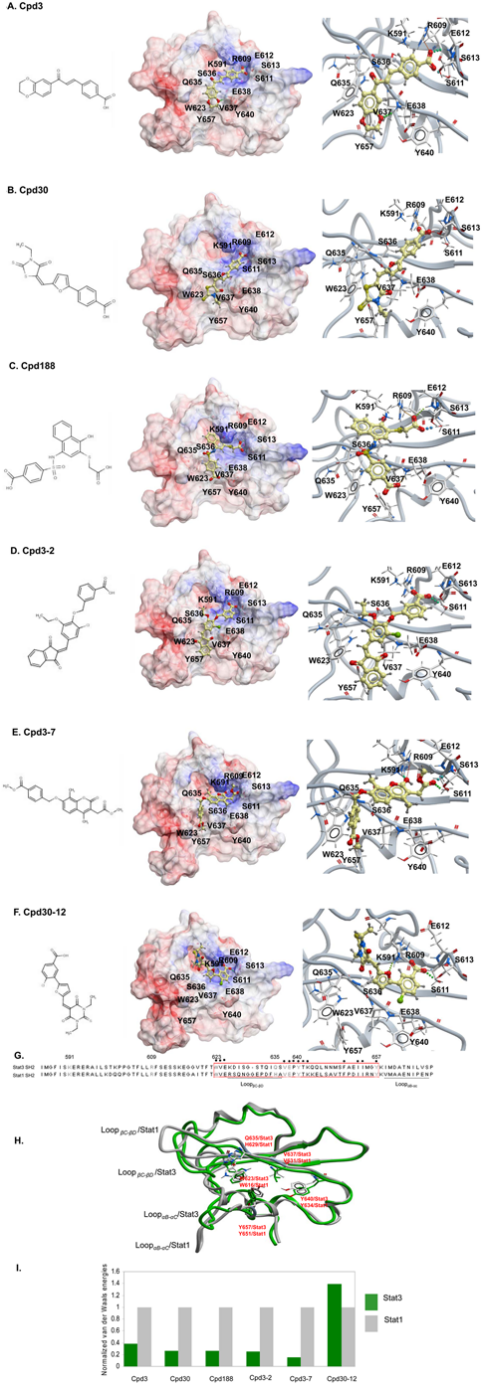
Computer modeling of each compound bound by the SH2 domain of Stat3 or Stat1. The 2-D structures and the results of computer docking of each compound to the Stat3 SH2 domain are shown in panels A through F. The left side of each panel shows the 2-D structure, the middle portion of each panel shows the compound binding to an electrostatic molecular surface model of the Stat3 SH2 domain in which blue represents areas of positive-charge and red represents areas of negative-charge. The right side of each panel is a closer view of this interaction with hydrogen bonds indicated by dotted lines. Stick models are used to depict critical residues in the general binding site (R609, K591, S611, E612 and S613), in the specific binding site (E638) and in the hydrophobic site (W623, Q635, V637, Y640 and Y657) with carbon, oxygen, nitrogen and hydrogen atoms represented by silver, red, blue and grey, respectively. Each compound is depicted using a ball-and-stick model with carbon, oxygen, nitrogen, sulfur, chlorine and hydrogen atoms represented by gold, red, blue, yellow and green, respectively. In panel A, the negatively charged benzoic acid moiety of Cpd3 has electrostatic interactions with the guanidinium cation group of R609 and the basic ammonium group of K591. There are double H-bonds that form between the carboxylic oxygen and the side chain terminus hydrogen of R609 and the amide hydrogen of E612 and H-bond formation between the benzoic acid carbonyl oxygen and the side chain hydroxyl hydrogen of S611. The oxygen atom of 1,4-benzodioxin forms a hydrogen bond with the amide hydrogen of E638. In addition, the double ring group of Cpd3 has hydrophobic interactions to the hydrophobic binding site, which consists of W623, Q635, V637, Y640, and Y657. In panel B, the carboxylic terminus of the benzoic acid moiety of Cpd30 has electrostatic interactions with the to guanidinium group of R609. There are two hydrogen bonds that form between the terminal hydrogen of R609 and carboxylic oxygen of Cpd30 and between the terminal hydrogen of S613 and carbonyl oxygen of Cpd30. In addition, the thiazolidin moiety of Cpd30 has hydrophobic interactions with the hydrophobic binding site. In panel C, the (carboxymethyl) thio moiety of Cpd188 has electrostatic interactions with R609 and K591. The terminal oxygen of the (carboxymethyl) thio group of Cpd188 forms three H-bonds: 1) with the guanidinium hydrogen of R609, 2) with the backbone amide hydrogen of E612 and 3) with the hydroxyl-hydrogen of S611. There is an H-bond formation between the hydroxyl-oxygen of the benzoic acid group of Cpd188 and the amide-hydrogen of E638. In addition, the benzoic acid group interacts with the hydrophobic binding site, particularly V637. In panel D, the benzoic acid group of Cpd3-2 has electrostatic interactions with R609 and K591. There are two H bonds between the carboxylic oxygen of the benzoic acid group and guanidinium hydrogen of R609 and between the carbonyl oxygen of the benzoic acid group and the hydroxyl hydrogen of S611. In addition, the 1,3-dihydro-2H-inden-2-ylidene group of Cpd30 has hydrophobic interactions with the hydrophobic binding site. In panel E, H-bond formation occurs between the carbonyl-oxygen of the benzoate moiety at the double-ring end of Cpd3-7 and the side chain hydroxyl hydrogen of S611 and the amide hydrogen of S613. H-bond formation also occurs the between the hydroxyl oxygen of Cpd3-7 and the guanidinium hydrogen of R609 and a hydrogen within the ammonium terminus of K591. In addition, the single ring group of Cpd3-7 has hydrophobic interactions with the hydrophobic binding site. In panel F, there are electrostatic interactions between the benzoic acid group of Cpd30-12 and R609 and K591. H-bond formation occurs between the carbonyl-oxygen of Cpd30-12 and the guanidinium-hydrogen of R609, between the carboxyl-oxygen of Cpd30-12 and the hydroxyl-hydrogen of S611 and between the furyl oxygen of Cpd30-12 and hydrogen within the ammonium terminus of K591. Panel G shows the sequence alignment of residues 585 to 688 of Stat3 and residues 578 to 682 of Stat1 each containing their respective SH2 domains. Residues K591, R609, S611, E612 and S613 that bind the pY residue are indicated in blue. Residue E638 that binds to the +3 residue is indicated in green. Residues W623, Q635, V637, Y640 and Y657 comprising the hydrophobic binding site are indicated in orange; the region within Stat3 and Stat1 that contains the hydrophobic binding site is boxed. Residues within Loop_βC–βD_ and Loop_αC–αD_ of Stat3 are each underlined. Residues identical between Stat3 and Stat1 are indicated by a dot. Panel H shows an overlay of tube-and-ribbon models of the SH2 domains of Stat3 (green) and Stat1 (gray). Residues within the hydrophobic binding surface of each are shown as stick models and Loop_βC–βD_ and Loop_αB–αC_ are indicated. The van der Waals energy of each compound bound to the Stat1 SH2 domain or the Stat3 SH2 domain was calculated, normalized to the value for Stat1 and shown in panel I.

To understand at the molecular level the basis for the selectivity of Cpds 3, 30, 188, 3-2 and 3-7 and the absence of selectivity in the case of Cpd 30-12, we compared the amino-acid sequences and the available structures of the Stat3 and Stat1 SH2 domains and also examined how each compound interacted with both. Sequence alignment revealed identity in the residues within Stat3 and Stat1 corresponding to the binding site for the pY-residue and the +3 residue (Figure 3G). In addition, overlay of the Stat3 and Stat1 SH2 structures revealed that the loops that contained these binding sites could be superimposed (Figure 3H). In contrast, sequence alignment revealed only 40% homology in the residues contained within the hydrophobic binding site from W623 to Y657 in Stat3 and the corresponding region of Stat1 (Figure 3G). Also, overlay of the Stat3 and Stat1 SH2 domain peptide backbone structures (Figure 3H) did not reveal superimposition throughout this region, particularly within Loop_βC–βD_ (K_626_DISGSTQIQS_636_), and the side chain of V637 in Stat3, which is pointed into the hydrophobic binding pocket, while the corresponding residue V631 in Stat1 is pointed away from the pocket. Review of computational models of Cpd3, Cpd30, Cpd188, Cpd3-2 and Cpd3-7 in a complex with the Stat3 SH2 domain revealed that each has significant interactions with the Stat3 SH2 domain binding pocket at all three binding sites, the pY-residue binding site, the +3 residue binding site and the hydrophobic binding site (Figure 3A, B, C, D, and E). In contrast, Cpd30-12 interacts with the pY-residue binding site and blocks access to the +3 residue-binding site but does not interact with or block access to the hydrophobic binding site (Figure 3F). In addition, while van der Waals energies of Cpd30-12 were equivalent for its interaction with the Stat3 SH2 domain and the Stat1 SH2 domain, the 5 selective compounds were much more favorable for their interaction with the Stat3 SH2 domain than with the Stat1 SH2 domain (Figure 3I). Thus, computer modeling indicated that activity of a compound against Stat3 derives from its ability to interact with the binding sites for the pY and the +3 residues within the binding pocket, while selectivity for Stat3 vs. Stat1 derives from the ability of a compound to interact with the hydrophobic binding site, which served as a selectivity filter. Van der Waals energy calculations (Figure 3I) implicated residues that form the hydrophobic binding site (W623, Q635, V637, Y640 and Y657) as critical for this selectivity. However, as noted previously, there is low homology between the Stat3 SH2 domain from residues W623 to Y657 that contain the hydrophobic binding site, and the corresponding residues in Stat1. Several of these non-homologous residues are polar residues raising the possibility that polar interactions of compounds within this region may also contribute to selectivity.

### Inhibition of nuclear translocation of phosphorylated Stat3 by Cpd3, Cpd30, Cpd188, Cpd3-2 and Cpd3-7 assessed by HTFM

**Figure 4:**
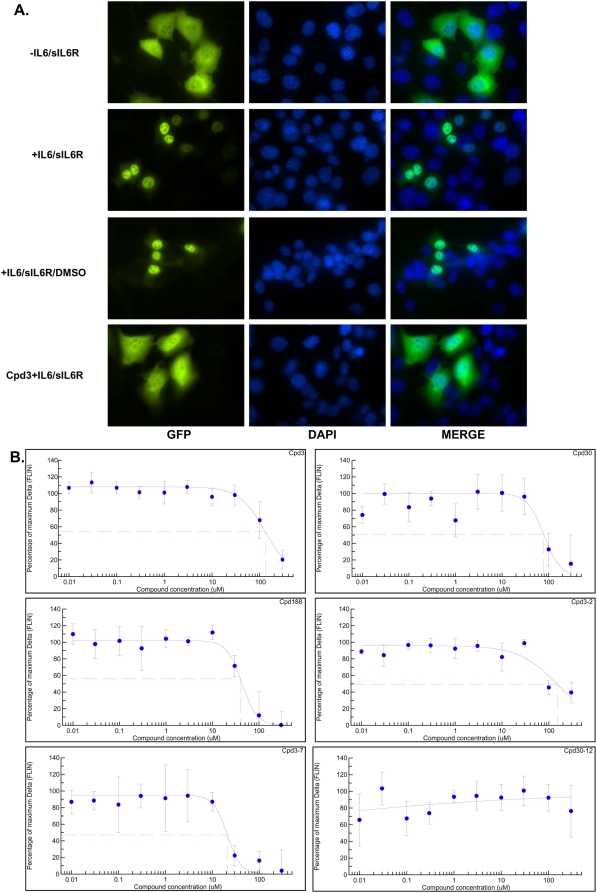
Inhibition of cytoplasmic-to-nuclear translocation of Stat3 assessed by confocal and high-throughput fluorescence microscopy (HTFM). In panel A, MEF/GFP-Stat3 cells grown on coverslips were pretreated with DMSO that either contained (row four) or did not contain (row three) Cpd3 (300 µM) for 60 min before being stimulated without (row one) or with IL-6 (200 ng/ml) and IL-6sR (250 ng/ml) for 30 minutes (rows two, three and four). Coverslips were examined by confocal fluorescent microscopy using filters to detect GFP (column one), DAPI (column two) or both (merge; column three). In panel B, MEF-GFP-Stat3 cells were grown in 96-well plates with optical glass bottoms and pre-treated with the indicated compound at the indicated concentrations in quadruplicate for 1 hour then stimulated with IL-6 (200 ng/ml) and IL-6sR (250 ng/ml) for 30 minutes. Cells were fixed and the plates were examined by high-throughput microscopy to determine the fluorescence intensity in the nucleus (FLIN). The percent of maximum change (delta, Δ) in FLIN was calculated as described in the Methods and plotted as a function of the log of the compound concentration. Data shown are mean±SD and are representative of 2 or more experiments. Best-fit curves were determined using 4-Parameter Logistic Model/Dose Response/XLfit 4.2, IDBS software and were used to calculate the IC_50_ values (Table 2).

Following its phosphorylation on Y705, Stat3 undergoes tail-to-tail dimerization mediated by reciprocal SH2/pY705-peptide ligand interactions. This conformational change is followed by nuclear accumulation. Compounds that targeted the Stat3 SH2/pY-peptide ligand interaction would be expected to inhibit nuclear accumulation of Stat3. To determine if this was the case with our compounds, we employed a nuclear translocation assay (Figure 4) using murine embryonic fibroblast (MEF) cells that are deficient in endogenous Stat3 but constitutively express GFP-tagged Stat3α at endogenous levels, MEF/GFP-Stat3α [31]. Preincubation of MEF/GFP-Stat3α cells with Cpd3, Cpd30, Cpd188, Cpd3-2 and Cpd3-7, but not Cpd30-12, blocked ligand-mediated nuclear translocation of GFP-Stat3α with IC_50_ values of 131, 77, 39, 150 and 20 µM respectively (Figure 4 and Table 2).

### Induction of apoptosis of breast cancer cell lines by Cpd3, Cpd30 and Cpd188; apoptosis is selective for cell lines with constitutive Stat3 activation

Previously identified compounds that target Stat3 induce cancer cell apoptosis [16]–[18], [20], [35]. To determine if any of the selective Stat3 compounds induce apoptosis and whether or not apoptosis induction is selective for tumor cell lines with constitutive Stat3 activation, we examined each of our Stat3 selective compounds for the ability to induce apoptosis of breast cancer cell lines, MDA-MB-231 [36]–[38], MBA-MB-468 [33], [39], [40] and MDA-MB-435 [33], [39] with constitutively active Stat3 and two breast cancer cell lines, MDA-MB-453 [17], [33], [39] and MCF7 [17], without constitutively active Stat3.

**Figure 5:**
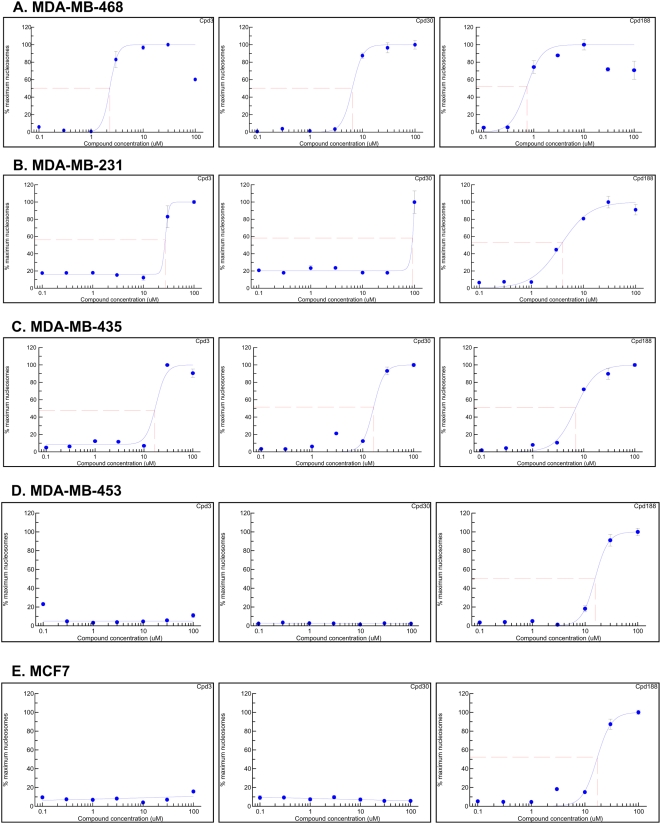
Apoptosis induction of breast cancer cell lines by compounds; selective apoptosis of cell lines that are Stat3 dependent. MDA-MB-468 (panel A), MDA-MB-231 (panel B), MDA-MB-435 (panel C), MCF7 (panel D) and MDA-MB-453 (panel E) were seeded in 12-well plates, grown overnight then treated with the indicated compound for 24 hr. Cells were centrifuged and the supernatants assayed for nucleosome levels by ELISA. The percent maximum nucleosome level was calculated (nucleosome level ÷ maximum nucleosome level achieved in the assay ×100) and plotted as a function of the log compound concentration. The best-fitting curve was determined using 4-ParameterLogistic Model/Dose Response One Site/XLfit 4.2, IDBS software and was used to determine the EC_50_ value (Table 3).

Two compounds—Cpd3 and Cpd30—induced apoptosis of the three breast cancer cell lines with constitutive Stat3 activity—MDA-MB-468, MDA-MB-231 and MDA-MB-435 (Figure 5A, B and C)—with EC_50_ values ranging from 2.3 to 26.9 µM and from 6.4 to 92.2 µM, respectively (Table 3). In contrast, neither compound induced apoptosis of cell lines MDA-MB-453 and MCF7 that do not demonstrate constitutive Stat3 activity in concentrations up to 300 µM (Figure 5D and E and Table 3). Cpd188 was even more effective than Cpd3 and Cpd30 at inducing apoptosis of cell lines with constitutive Stat3 activity (Figure 5A, B and C and Table 3) demonstrating EC_50_ values ranging from 0.7 to 7 µM (mean±SD = 3.9±3.1 µM). Unlike Cpd3 and Cpd30, however, Cpd188 also had detectable activity against MDA-MB-453 and MCF7 (Figure 5D and E and Table 3), demonstrating EC_50_ values ranging from 17.2 to 15.5 µM, respectively (mean±SD = 16.4±1.2). Nevertheless, comparison of the EC_50_ values of Cpd188 for the two groups of breast cancer cell lines indicated that, similar to Cpd 3 and Cpd30, Cpd188 showed preferential activity against cell lines with constitutive Stat3 activity (p = 0.014, Student's t-test). In contrast to Cpd3, Cpd30 and Cpd188, neither Cpd3-2 nor Cpd3-7 induced apoptosis of any of the breast cancer cell lines tested (data not shown).

**Table 3:** Summary of activity of compounds that are selective for Stat3 in inducing apoptosis of breast cancer cell lines.

Cell line	Cpd3	Cpd30	Cpd188	CAM^1^
**MDA-MB-468**	2.28^2^	6.42	0.73	0.74
**MDA-MB-231**	26.91	92.01	3.96	1.62
**MDA-MB-435**	16.5	16.29	7.01	1.64
**MCF7**	>300	>300	17.19	0.13
**MDA-MB-453**	>300	>300	15.5	0.93

1 CAM, camptothecin.

2 Data presented are EC_50_ values (µM) calculated from results summarized in Figure 5.

## Discussion

To develop chemical probes that selectively target Stat3, we virtually screened 920,000 small drug-like compounds by docking each into the pY-peptide-binding pocket of the Stat3 SH2 domain, which consisted of three sites—the pY binding site, the +3 residue-binding site and a hydrophobic binding site. Three compounds satisfied criteria of interaction analysis, inhibited recombinant Stat3 binding to its immobilized pY-peptide ligand and inhibited IL-6-mediated tyrosine phosphorylation of Stat3. These compounds were used in a similarity screen of 2.47 million compounds, which identified 3 more active compounds. Examination of the 6 positive compounds for the ability to inhibit IFN-γ-mediated Stat1 phosphorylation revealed that 5 of 6 were selective for Stat3 vs. Stat1. Sequence and structural analysis of the SH2 domains of Stat3 and Stat1 revealed that the ability of the compound to interact with the hydrophobic binding site was the basis for selectivity. All 5 selective compounds inhibited nuclear-to-cytoplasmic translocation of Stat3, while 3 of 5 preferentially induced apoptosis of breast cancer cell lines with constitutive Stat3 activation with one compound (Cpd188) active against one breast cancer cell line (MDA-MB-468) in the sub-micromolar range. Thus, virtual ligand screening of compound libraries targeting the pY-peptide binding pocket of the Stat3 SH2 domain identified for the first time 3 lead compounds that competitively inhibit Stat3 SH2 domain binding to its pY-peptide ligand, selectively target Stat3 vs. Stat1 and induce apoptosis preferentially of breast cancer cells lines with constitutively activated Stat3.

Several molecules have been identified recently that target Stat3 [15]–[20], [41]–[44]. Fluorescence polarization studies indicated that a peptidomimetic, hydrocinnamoyl-Tyr(PO3H2)-Leu-cis-3,4-methanoPro-Gln-NHB, was a potent inhibitor of Stat3 binding to pY-peptide binding with an IC_50_ of 125 nM [15]. Results of its ability to inhibit Stat3 phosphorylation or nuclear translocation within cells have not been reported reflecting, perhaps, the general obstacle of cell permeability posed by the peptidomimetic class of drugs.

The G-rich, quartet-forming oligodeoxynucleotide, T40214, was identified as a Stat3 inhibitor through docking studies of T40214 onto the known structure of Stat3 [45]. T40214 targeted Stat3 tail-to-tail homodimers, decreased Stat3 binding to DNA and inhibited growth of prostate, breast and lung cancer cells in the nude mouse xenograft model through induction of apoptosis [20], [35], [45]–[47]. T40214 is administered IV or intra-peritoneally in a complex with polyethyleneimine, which greatly improves intracellular uptake. To complement these efforts by our group and to develop a different class of Stat3 inhibitor for use in cancer treatment with the potential for oral administration, we determined if recent information obtained regarding the structural requirements of Stat3 SH2/pY-peptide binding [23], [27] could be exploited to develop a small-molecular inhibitor of Stat3.

Other groups have taken a small-molecule approach to targeting Stat3 with some success. STA-21 is a small molecule inhibitor of Stat3 identified through virtual ligand screening of compounds that bound to the interface of Stat3 SH2 homodimers [17]. STA-21 treatment of cells disrupted Stat3/DNA complexes, abrogated Stat3 translocation into the nucleus, inhibited expression of proteins such as Bcl-_X_L and Cyclin D1 and induced the apoptosis of breast cancer cell lines. No evidence was provided that STA-21 bound directly to Stat3 reflecting, perhaps, the non-availability of suitable reagents i.e. purified Stat3 homodimers. More recently, a model of STA-21 interaction with the Stat3 SH2 pY-peptide binding pocket has been proposed, which featured the 1-oxygen of STA-21 binding to the side chain ammonium hydrogen of R609 within the pY-residue binding site. Chemical modification of STA-21 was undertaken with the goal to generate compounds with improved interaction at this site. Four compounds were synthesized and the most potent of these demonstrated activity similar to STA-21 with an EC_50_ for apoptosis induction of three Stat3-dependent prostate cancer cell lines with constitutive Stat3 activity ranging from 13.4 to 34.1 µM [19].

Schust *et al.* [16] identified another small molecule inhibitor of Stat3, Stattic, using a fluorescence polarization high throughput assay of Stat3 binding. This group screened 17,298 chemical compounds and identified 144 compounds with significant activity in this assay. The most active compound, Stattic, inhibited Stat3 binding to a cognate pY-peptide ligand, inhibited ligand-mediated Stat3 phosphorylation and nuclear translocation, reduced Stat3 binding to DNA and induced apoptosis of breast cancer cells with constitutively activated Stat3 in the 5–20 µM range. Similar to the compounds we identified, inhibition of ligand-induced phosphorylation was selective for Stat3 vs. Stat1. Unlike our compounds, however, inhibition of Stat3 by Stattic was blocked by addition of a reducing agent (dithiothreitol, DTT), was not reversible, and may not be mediated by direct inhibition of pY-peptide binding. Rather, Stattic may alter the shape of the Stat3 SH2/pY-peptide binding site through alkylating the C687 residue on the opposite side of the SH2 domain [48].

Siddiquee *et al.* [18] recently identified a small molecule Stat3 inhibitor, S3I-201, using an approach similar to ours that targeted the Stat3 SH2 pY-peptide binding site. S3I-201 inhibited Stat3 homodimerization, DNA binding, induction of cyclin D1, Bcl-xL and survivin and induced apoptosis of v-Src-transformed NIH3T3 cells and breast cancer cell lines with constitutively active Stat3 in the 30 to 100 µM range. Similar to T40214, S3I-201 (5 mg/kg every 2–3 days) inhibited growth of nude mice xenografts of one of these breast cancer cell lines (MDA-MB-231). Similar to STA-21, but unlike the compounds we identified, no evidence of the ability of S3I-201 to directly bind Stat3 or to inhibit the binding of Stat3 to its pY-peptide ligand was presented leaving open the question of the precise mechanism of action of S3I-201.

The use of molecular modeling to delineate the structural basis for competitive inhibition of Stat3 SH2/pY-peptide binding by our compounds identified the hydrophobic binding site as a selectivity filter. Molecular modeling also provides a rational basis for modification of our three lead compounds to identify related ones with greater potency; these studies are underway. In addition, the strategy employed here can be used to develop selective chemical probes for other members of the Stat protein family. In addition to Stat3 and Stat1, structural information currently is available for Stat5A [49]. Overlay of the SH2 domains of Stat5A and Stat1 and of Stat5A and Stat3 revealed differences within the pY-peptide binding site of Stat5A and both Stat1 and Stat3. We are currently pursuing VLS screening to exploit these differences to develop selective chemical probes of Stat5 for use in chemical genomic studies and as potential therapy for cancers in which Stat5 contributes to oncogenesis.
